# Letter from E. S. Bennett, M. D.

**Published:** 1845-09

**Authors:** E. S. Bennett


### ARTICLE VII.

For the following letter we are indebted to Dr. J. A. Cleveland, of Charleston, S. C.—*Eds.*

Charleston, 16*th July*, 1845.

Early in the month of March, 1845, I was requested to visit, professionally, a negro child about thirty months old, the property of Col. J. L. of our city, who was reported to have a singular appearance about the mouth, resembling a piece of bone growing from and connected with the inferior maxillary, preventing the child from mastication, and it could only eat fluids, and that but sparingly. I found the child in an extremely emaciated condition—having been suffering for five months; upon examining it with some care, I found the bone (inferior maxilla) in a state of *necrosis*, extending from the canine tooth on the right side, along the whole bone on its entire aspect, to the articulation on the left. The anterior portion of the bone had been raised from its position natural, and becoming elevated as far back as the ramus on the left, and its point, which was rough and very rugged, fixed in the soft parts on the right side of the corner of the mouth, and from this state of irritation an extensive and frightful ulcer was developed. The only question suggesting itself was, whether the child, in its then emaciated condition, could survive the operation; the chances I considered as equal, and determined upon dilation of the soft adhering part within the mouth, and removing the whole mass, which was accordingly done, and with a pair of strong curved forceps, the bone was seized as far back as the bend, and by a careful rotary motion of the hand, the disarticulation was accomplished, and the bone removed. Within a few days, a decided and pleasing improvement was observed; and in four weeks, I was enabled to return the little sufferer to the country.

It may be asked, and with propriety, what was the exciting cause of so frightful state of things?—had the child taken mercury in any of its forms? I think I may safely say *not;* having been myself in attendance on the plantation for ten years, and can safely say none; but the disease may be, and probably was sui generis, or the result of some local hyperimia—the result of the process of dentition.

This case settles the question definitely as to whether nature of herself is capable of reproducing the bony structures entirely; in this case, not only the whole bone has been reproduced, but dentition also—being now armed with two formidable grinders.

Should this case prove, in your view, as interesting, you can use it as you think proper.

I am, dear sir, very respectfully, yours,

E. S. BENNETT, M. D.

To J. A. Cleveland, D. D. S.,
*Charleston, S. C.*

